# Oncology and Pharmacogenomics Insights in Polycystic Ovary Syndrome: An Integrative Analysis

**DOI:** 10.3389/fendo.2020.585130

**Published:** 2020-10-26

**Authors:** Verónica Yumiceba, Andrés López-Cortés, Andy Pérez-Villa, Iván Yumiseba, Santiago Guerrero, Jennyfer M. García-Cárdenas, Isaac Armendáriz-Castillo, Patricia Guevara-Ramírez, Paola E. Leone, Ana Karina Zambrano, César Paz-y-Miño

**Affiliations:** ^1^ Centro de Investigación Genética y Genómica, Facultad de Ciencias de la Salud Eugenio Espejo, Universidad UTE, Quito, Ecuador; ^2^ Latin American Network for the Implementation and Validation of Clinical Pharmacogenomics Guidelines (RELIVAF-CYTED), Madrid, Spain; ^3^ Centro de Atención Ambulatorio, Hospital del Día El Batán, Instituto Ecuatoriano de Seguridad Social (IESS), Quito, Ecuador

**Keywords:** polycystic ovary syndrome (PCOS), endometrial cancer (EC), ovarian cancer (OC), breast cancer (BC), pharmacogenomics, bioinformatic

## Abstract

Polycystic ovary syndrome (PCOS) is a heterogeneous endocrine disorder characterized by hyperandrogenism, ovulatory dysfunction, and polycystic ovaries. Epidemiological findings revealed that women with PCOS are prone to develop certain cancer types due to their shared metabolic and endocrine abnormalities. However, the mechanism that relates PCOS and oncogenesis has not been addressed. Herein, in this review article the genomic status, transcriptional and protein profiles of 264 strongly PCOS related genes (PRG) were evaluated in endometrial cancer (EC), ovarian cancer (OV) and breast cancer (BC) exploring oncogenic databases. The genomic alterations of PRG were significantly higher when compared with a set of non-diseases genes in all cancer types. *PTEN* had the highest number of mutations in EC, *TP53*, in OC, and *FSHR*, in BC. Based on clinical data, women older than 50 years and Black or African American females carried the highest ratio of genomic alterations among all cancer types. The most altered signaling pathways were p53 in EC and OC, while Fc epsilon RI in BC. After evaluating PRG in normal and cancer tissue, downregulation of the differentially expressed genes was a common feature. Less than 30 proteins were up and downregulated in all cancer contexts. We identified 36 highly altered genes, among them 10 were shared between the three cancer types analyzed, which are involved in the cell proliferation regulation, response to hormone and to endogenous stimulus. Despite limited PCOS pharmacogenomics studies, 10 SNPs are reported to be associated with drug response. All were missense mutations, except for rs8111699, an intronic variant characterized as a regulatory element and presumably binding site for transcription factors. In conclusion, *in silico* analysis revealed key genes that might participate in PCOS and oncogenesis, which could aid in early cancer diagnosis. Pharmacogenomics efforts have implicated SNPs in drug response, yet still remain to be found.

## Introduction

### PCOS Diagnose Criteria

Polycystic ovary syndrome (PCOS) is a heterogeneous endocrine disorder characterized by hyperandrogenism, ovulatory dysfunction, and polycystic ovaries, that affects reproductive aged women. The phenotype of this syndrome is a complex interplay between genes, proteins, epigenetics, and environmental factors (1). Clinical manifestations of PCOS include irregular menstruation, infertility, hirsutism, acne, and the metabolic syndrome (2).

In 1990 the National Institutes of Health (NIH) indicated that PCOS diagnostic criteria included oligo-anovulation/anovulation, and clinical/biochemical signs of hyperandrogenism. Efforts have been made to redefine the PCOS diagnosis guidelines; in 2003, the European Society of Human Reproduction and Embryology and the American Society for Reproductive Medicine (ESHRE/ASRM), defined the Rotterdam criteria, adding ultrasound examination of polycystic ovaries to the previous traits. According to the Rotterdam criteria, women are diagnosed with PCOS if at least two traits are met (3). In 2006, The Androgen Excess and PCOS Society (AES) reviewed all the reports about PCOS to characterize its phenotype. Although no additional features were added, the report emphasized hyperandrogenism as a decisive medical condition for PCOS diagnosis (4). All aforementioned criteria exclude androgen excess disorders, such as congenital adrenal hyperplasia, Cushing’s syndrome, androgen-secreting tumors, hyperprolactinemia, thyroid diseases.

Based on the phenotype, PCOS patient can be classified in four sub phenotypes accepted by the Rotterdam criteria. A classical PCOS (phenotypes A and B) grouped women with the three criteria and women without polycystic ovary but oligo-ovulation, and hyperandrogenism. Women in group C ovulate and have a combination of polycystic ovaries and hyperandrogenism. In group D non hyperandrogenic patients present polycystic ovaries and oligo-ovulation. NIH criteria accepted only the classical PCOS phenotype while AES only phenotypes A, B and C (5).

## Oncology and PCOS

### PCOS and Cancer Risk

Fourteen years after the syndrome was firstly described, Speert (6) noted a recurrent presence of cystic ovaries in young women (< 40 years old) with endometrial cancer (EC). In 1957, this observation was examined in 43 available biopsies from obese, hypertensive women with irregular menstruations, and signs of hyperandrogenism. Of them 16 had endometrial carcinoma (7). The results of the first evaluation of breast cancer (BC) risk and polycystic ovaries, in 1991, revealed a low cancer risk in women with polycystic ovarian morphology (odds ratio [OR], 0.52; 95% confidence interval [CI], 0.32–0.87) (8). Lastly, ovarian cancer (OC) and its association with women diagnosed with PCOS were evaluated in 1996. It was found a 2.5 fold increased risk of OC among them (95% CI, 1.1–5.9) (9).

A vast number of reports exploring PCOS and cancer risk have been published using diverse study design types such as case series, case reports, case-control, cohort and cross-sectional studies (10). The criteria for patient selection, the limited number of cases recruited, and the lack of consideration of confounders led to contradictory evidence of cancer and PCOS. Due to these limitations, Hardiman et al. (11) stated that a meta-analysis to estimate the relative risk of EC could not be performed. On the contrary, three meta-analysis that evaluated the association between EC, OC, and BC were published (12–14). The number of studies included in each meta-analysis are varied and some repetitively considered (**Table 1**). Studies selected for these meta-analysis had cohort information of PCOS and non-PCOS patients who developed or not cancer. PCOS diagnosis was not a restrictive selection criteria and self-reports were accepted. Only 1 out of 14 studies used Rotterdam criteria for PCOS diagnosis (25). EC, OC and BC were histologically confirmed and combined for the analysis, regardless clinical stage or histological type. Diverse cohorts from several geographical locations were included for these meta-analysis, so the results have a large-scale health-impact.

**Table 1:** Meta-analysis that detect the risk of gynecological cancer and PCOS.

Cancer type	No. studies (methodology)	No. studies PCOS criteria	No. PCOS patients* (sample size)	Age range	Cohort origin or ethnicity	Individual study OR (95%CI)	Study reference	Meta-analysis, OR (95%CI) at age range
Chittenden et al. (12)								
Endometrial cancer (EC)	3 case- control 1 cross-sectional retrospective	2 NS 1 Goldzieher 1 Particular traits	56 (4,056)	<40–69	USA	5.4 (2.4–12.3)	(15)	2.70 (1.00–7.29)
					100% Japanese	8.9 (0.4–184.9)^b^	(16)	
					66% Caucasian, 2% Asian-Indian, 1% Asian other, 2% African-Caribbean, 29% unreported	1.0 (0.4–2.7)^b^	(17)	
					Greek	9.0 (0.5–176.0)^b^	(18)	
Ovarian Cancer (OC)	1 case- control	NS	31 (4,547)	20–54	USA	2.5 (1.1–5.9)^⊗^	(9)	2.52 (1.08–5.89)
Breast Cancer (BC)	3 case-control	3 NS	133 (23,842)	20–75	USA	0.5 (0.3–0.9)^⊗^	(8)	0.89 (0.44–1.77)
					Italy	0.8 (0.4–1.7) ^⊘^	(19)	
					USA	1.6 (0.8–3.2) ^⊘^	(20)	
Haoula et al. (13)								
EC	4 case- control 1 cross-sectional retrospective	3 NS 1 Goldzieher 1 Particular traits	88 (4,605)	<40–69	Australia plus EC studies in Chittenden et al. (12)	2.2 (0.9–5.7) ^ϕ^	(21)	2.89 (1.52–5.48)
Barry et al. (14)								
EC	5 case-control	4 NS 1 Goldzieher	138(5731) 70 (4376)^a^	18–79 20–54^a^	USA	5.4 (2.4–12.3)	(15)^a^	2.79 (1.31–5.95) 4.05 (2.42–6.76)^a^
					100% Japanese	8.9 (0.4–184.9)^b^	(16)^a^	
					Greek	9.0 (0.5–176.0)^b^	(18)^a^	
					Italy	1.25 (0.72–2.16) ^ϕ^	(22)	
					Australia	2.2 (0.9–5.7) ^ϕ^	(21)^a^	
OC	3 case- control	2 NS	111 (18489) 31 (4557)^a^	18–79 20–54^a^	USA	2.5 (1.1–5.9)^⊗^	(9)^a^	1.41 (0.93–2.15) 2.52 (1.08–5.89)^a^
					Australia	1.1 (0.6–2.0) ^⊘^	(23)	
					United Kingdom	1.63 (0.65–4.08)	(24)	
BC	2 case-control 1 cohort	2 NS 1 Rotterdam	529 (40324) 57 (5489)^a^	20–74 20–54^a^	Italy	0.8 (0.4–1.7) ^⊘^	(19)^a^	0.95 (0.64–1.39) 0.78 (0.46–1.32)^a^
					Iran	0.66 (0.299–1.48)	(25)^a^	
					USA	1 (0.6–1.9) ^⊘ ∇^	(26)	

*Number of PCOS patient among cancer cases and controls, CI, confidence interval; NS, not stated, ^⊗^Age adjusted, ^⊘^Adjusted for multiple variables: age, education, parity, body mass index among others, ^ϕ^Body Mass Index adjusted, ^a^EC in PCOS younger than 54 years, ^∇^Risk ratio, ^b^OR calculated with data provided in the article.

The evidence provided by these meta-analysis suggested that pre/post-menopausal women diagnosed with PCOS are nearly three times more likely to develop EC than women without this endocrinopathy (12, 13). The association is even emphasized when the analysis is limited to women aged < 54 years old (OR, 4.05; 95% CI, 2.42–6.76) (14). Regarding OC, women with PCOS and under the age of 54, have a 2.5 fold increased risk of develop OC. Women with this disorder seem not to be under risk of BC (12, 14).

Recent studies evaluated PCOS as a disorder that predispose women to other cancer types. A large Danish cohort reported that women with PCOS have developed several types of malignant neoplasm. Endometrium and kidney observed cases were nearly four times higher than the predicted ones. Brain (standardized incidence ratio [SIR], 2.2; 95% CI, 1.3–3.5) and colon tumor risks (2.1; 95% CI, 1.1–3.8) were significantly elevated, in contrast to BC and OC without significant increased risk (27). Another cohort from Sweden found an increased overall cancer risk (hazard ratio [HR], 1.15; 95% CI, 1.00–1.33), being endometrium, ovary, endocrine gland, kidney, skeletal, and hematopoietic system the sites where the risk was considerably elevated. Cancer risk was predominantly restricted among premenopausal women, as reported in the meta-analysis studies aforementioned (28).

EC has been classified into two types: Type I comprises endometroid adenocarcinomas that initiate from endometrial hyperplasia and are estrogen-dependent. Type II tumors are estrogen-independent serous carcinoma and have worse prognosis than Type I (29). Type I tumors are the most commonly reported in PCOS patients (2). Indeed a higher risk of PCOS and Type I endometrial cases was noted (OR, 2.4; 95% CI, 1.0–6.2) compared with unclassified EC cases (OR, 2.2; 95% CI, 0.9–5.7). OC is classified in four histological subtypes: serous, clear cell, endometrioid, mucinous. Olsen et al. (23) have reported a significant association of serous borderline tumors and PCOS (OR, 2.6; 95% CI, 1.0–6.1). Another study conducted by Harris et al. (30) detected a significant reduced risk of ovarian serous tumors in women with menstrual cycle irregularity. Based on the location, BC types are ductal and lobular carcinoma and they can be invasive or non-invasive (31). According to the expression of estrogen receptor (ER), progesterone receptor (PR), and human epithelial growth factor receptor 2 (HER2), breast cancer can be classified into four categories: HER2−, ER+ and/or PR+; HER2+, ER+ and/or PR+; HER2+, ER−, PR−, and triple negative (HER2−, ER−, and PR−) (32). To our knowledge there are no reported studies that have integrated BC types and PCOS cancer risk.

Several PCOS factors such as nulliparity, obesity, and hormonal imbalances are associated with cancer (27). Briefly, chronic mitogen stimulation *via* estrogen in the endometrium unopposed by the inhibitory effects of progesterone, overproduction of luteinizing hormone (LH), upregulation of aromatase activity, elevated insulin-like factor-1 (IGF-1) concentrations, and insulin resistance (IR) may represent risk factors for EC (10, 33). Body mass index (BMI), age, and contraception intake are noted to affect the strength of association between OC and PCOS (9, 21). Since androgens are precursors of estrogens, androgen excess might lead to estrogen overproduction and therefore breast cell proliferation. Besides, infertility, IR, and obesity are comorbidities associated with BC risk (34).

### PCOS and Cancer Genetics

Genomic, transcriptomic, proteomic, and epigenomic studies in PCOS are shedding light on the molecular basis and the biological mechanism of the PCOS pathogenesis (35). Gene expression profiles analysis in conjunction with bioinformatic tools have been widely used to identify characteristic patterns of genes expression, distorted biological pathways, gene interactome, and even drug signatures to treat PCOS (36–39). Yet, reduced number of samples and varied tissue types (adipose tissue, theca cells, granulosa cells, cumulus cells, endothelial cells) were included in the *in silico* approaches.

Regarding proteomic biomarker profiling in PCOS, there are two studies that have compiled a list of 180 biomarkers from an integrative literature review (40, 41). Nine proteins (TAGLN, PKM2, CAPG, GSTP1, LAP3, FKBP3, PPIA, C4A, and SOD2) had the same pattern of expression in PCOS and EC samples in serum and endometrium.

In relation to cancer, a research work that isolated endometrial cell populations from women with PCOS revealed enhanced expression of cytokines and immune response genes in several cell populations. Particularly endothelial and mesenchymal stem cells displayed changes in inflammatory and cancer related genes (42). Kori et al. (43) identified that PCOS, endometriosis and OC shared common signatures by an integrative transcriptomic data analysis. This study identified that pathways in cancer (MAPK signaling pathways) and cellular functions (cell cycle and apoptosis) were enriched terms in the three diseases and might collectively contribute to their clinical course. Another research using PCOS related proteins noted that OC, endometriosis, and other 15 diseases significantly shared proteins and pathways (44). In a survival analysis, after examining PCOS differentially expressed genes (DEG) in patients with ovarian serous cystadenocarcinoma, a correlation with disease-free survival and overall survival was found. This finding highlighted the critical role of these genes in PCOS and its long term complications, particularly in OC (45). Even in pathway enrichment analysis of differentially methylated genes participating in a protein-protein interaction network, pathways associated with cancer, chronic myeloid leukemia, and prostate cancer were mainly enriched (46). An additional study described that women with PCOS and irregular menstruation displayed DNA hypomethylation, specifically in oncological relevant sites. Also, the patients with irregular menstruation and OC had comparable expression profiles of onco-miRNA and cancer related-genes, indicating that irregular menstruation is a risk factor for OC (47).

Although endometrium is the major pathologically targeted tissue, few studies have considered PCOS endometrium with and without endometrial hyperplasia to compare their gene and/or protein expression. Moreover, their analysis have focused on a reduced number of targeted genes. Villavicencio et al. (48) studied the expression of steroid receptors and their coregulators in PCOS endometrium with (n=7) and without hyperplasia (n = 6–5) by RT-PCR, western blot and immunostaining. Higher expression of androgen receptor and estrogen receptor β was found in PCOS endometrium with hyperplasia when compared with normal endometrium and women with PCOS, respectively. Activators also were highly expressed, being *ARA70* elevated in PCOS endometrium with hyperplasia, denoting a key role in steroid responsiveness, which in turn control cell cycle. The sterol regulatory element binding protein 1 (*SREBP1*) regulates lipid synthesis and *in vitro* assays evidenced that this gene supports cancer cell growth and proliferation. Alongside, a significant increase in *SREBP1* expression, was noticed in PCOS (n=34) and endometrial cancer (n=34) endometrium using RT-PCR (49). In addition, Wang et al. (50) studied protein expression patterns of glycolytic enzymes, androgen receptor, estrogen receptor and mitochondria related components, due to their known partaking in this syndrome and in the development of metabolic diseases. They reported a decreased glycolysis and increased mitochondrial activity in PCOS patients with endometrium hyperplasia, profusely expressing estrogen receptor α (n=7).

This evidence highlights the importance of additional research integrating diverse perspectives to clarify the relationship between cancer and PCOS. Independently, the genetic research in PCOS and cancer has generated large amount of information which has been deposited in databases. Hereafter we combine both diseases data to examine the status of PCOS-related genes, as proxy of the syndrome, in EC, OC, and BC.

### Bioinformatic Exploration

A list of PCOS related genes (PRG) were created based on literature review and genomic platforms such as DisGeNET (51), Ensembl (52), PCOSKB (53) DISEASES (54). Only 264 strongly associated genes, based on Open Targets Platform disease score, (55) were selected for further analysis (**Supplementary Data**) (**Supplementary Table 1**).

#### Enrichment Map of Associated PCOS Genes

**Figure 1:**
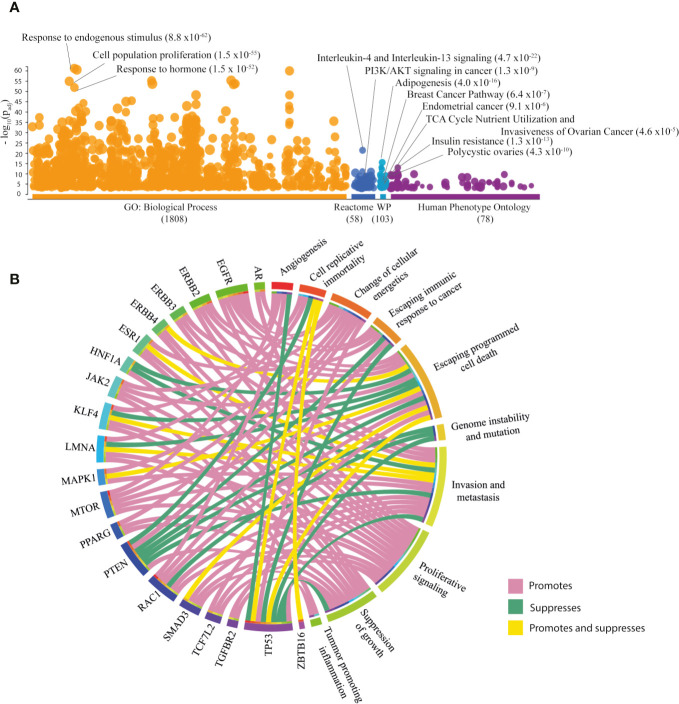
Exploration of associated PCOS genes (PRG [n=264]). **(A)** Most significant GO: biological processes, Reactome pathways, WikiPathways (WP) and Human Phenotype Ontology according to g:Profler Manhattan plot. **(B)** Circos plot of PRG with hallmarks of cancer taken from COSMIC database.

To investigate the biological and clinical role of the 264 PRG, g:profiler was accessed and the results are illustrated in the Manhattan plot (**Figure 1A**) (56). The most significant (FDR < 0.001) Gene Ontology (GO) term was response to endogenous stimulus (**Supplementary Table 2**). Interleukin-4 and interleukin-13 signaling was the most significant pathway in Reactome, while adipogenesis was the most significant term in WikiPathways (**Supplementary Table 3**). The most relevant diseases in the Human Phenotype Ontology were IR, and polycystic ovaries (**Supplementary Table 4**). Other relevant terms relating to cancer are shown in **Figure 1A**.

#### Associated PCOS Genes and Hallmarks of Cancer

The Catalogue of Somatic Mutations in Cancer (COSMIC) served as a platform to search for hallmarks of cancer in the PRG. Of the 264 PRG, 20 (7.58%) genes were correlated with cancer hallmarks (**Figure 1B**) (57). The key hallmarks of cancer that grouped the highest number of genes were invasion and metastasis, promoted by *EGFR*, *ERBB2*, *ERBB3*, *MAPK1*, *MTOR*, *RAC1*, *SMAD3*, *TCF7L2*, and *TGFBR2*; escaping cell death, promoted by *EGFR*, *ERBB2*, *ERBB3*, *ERBB4*, *JAK2*, *MTOR*, and *TCF7L2*. Additionally, *TP53* followed by *PTEN*, *EGFR*, and *RAC1* were genes enriched in hallmarks of cancer (**Supplementary Table 5**).

#### Overview of Genomic Alterations of PRG in TCGA PanCancerAtlas

cBioPortal has reported clinical and genomic data from more than 20 cancer studies, for the purpose of this report information from uterine corpus endometrial carcinoma (n=507), ovarian serous cystadenocarcinoma (n=201), and breast invasive carcinoma (n=994) TCGA PanCancerAtlas studies were retrieved (**Table 2**) (58, 59). To evaluate the genomic status of PRG, the frequency of genomic alterations in PRG was compared with a PCOS non related gene set (PNRG) separately in EC, OC, and BC (**Supplementary Table 1**). The PNRG were selected from a list of non-disease genes rationally filtered (60).

**Table 2:** Description of the individuals for genomic alteration analysis.

Age	Endometrial Cancer		Ovarian Cancer		Breast Cancer	
	N°	%	N°	%	N°	%
≤ 50	45	8.88	47	23.38	299	30.08
>50	459	90.53	143	71.14	695	69.92
Unknown	3	0.59	11	5.47	0	0.00
Total	507	100	201	100	994	100
**Race**						
	**N°**	**%**	**N°**	**%**	**N°**	**%**
American Indian or Alaska Native	4	0.79	2	1.00	1	0.10
Asian	20	3.94	7	3.48	59	5.94
Black or African American	101	19.92	19	9.45	162	16.30
Native Hawaiian or Other Pacific Islander	9	1.78	0	0	0	0
White	342	67.46	157	78.11	687	69.11
Unknown	31	6.11	16	7.96	85	8.55
Total	507	100	201	100	994	100

N, number of individuals; %, percentage.

**Figure 2:**
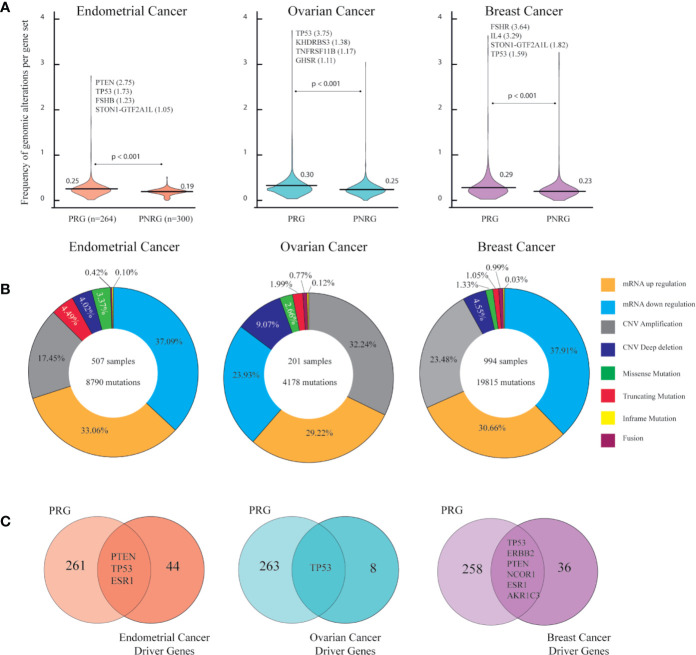
Genomic alterations in endometrial, ovarian and breast cancer according to PanCancer Atlas. **(A)** Frequency of genomic alterations per gen set (associated PCOS genes (PRG [n=264]) and not associated PCOS genes (PNRG [n=300]) in each cancer type. Mann-Whitney U test with significant level of p < 0.05. **(B)** Percentage of genomic alterations types in each cancer type. **(C)** Identification endometrial, ovarian and breast cancer driver genes found in the list of PRG.

**Figure 2A** shows the normalized frequency mean of genomic alterations from the two gene sets per cancer type. The frequency mean of the PRG in OC was 0.30 followed by BC (0.29) and EC (0.25). In all cancer types, statistical test evidenced a significantly (p < 0.001) higher frequency of genomic alterations in PRG than in PNRG (**Supplementary Tables 6–8**). As expected a multiple comparison between PRG, PNRG, and well known-cancer driver genes in each cancer types showed a higher frequency of genomic alterations in cancer driver genes, followed by PRG with a significant Bonferroni correction of p < 0.001 (data not shown). The tumor suppressor gene, *PTEN*, showed the highest number of mutations in EC. The well-known cancer driver gene, *TP53*, was the top hit in OC, whereas *FSHR* in BC (**Figure 2A**). **Figure 2B** presents the percentage of all genomic alterations in EC, OC, BC. The most common alteration in EC (37.09%) and BC (37.91%) was related to mRNA downregulation, while in OC, gene amplifications (32.24%) were more frequent than any mRNA alterations (**Supplementary Table 9**). It is worth mentioning, that among PRG, there were driver genes: *PTEN*, *TP53*, *ESR1* for EC, *TP53* for OC, and *TP53*, *ERBB2*, *PTEN*, *NCOR1*, *ESR1*, *AKR1C3* for BC (**Figure 2C**).

From this first omics approach we obtained a lists of genes with the highest number of genomic alterations (above the frequency mean) from EC (n=94), OV (n=96), and BC (n=61).

#### Exploration of Clinical Features in TCGA PanCancerAtlas

**Figure 3:**
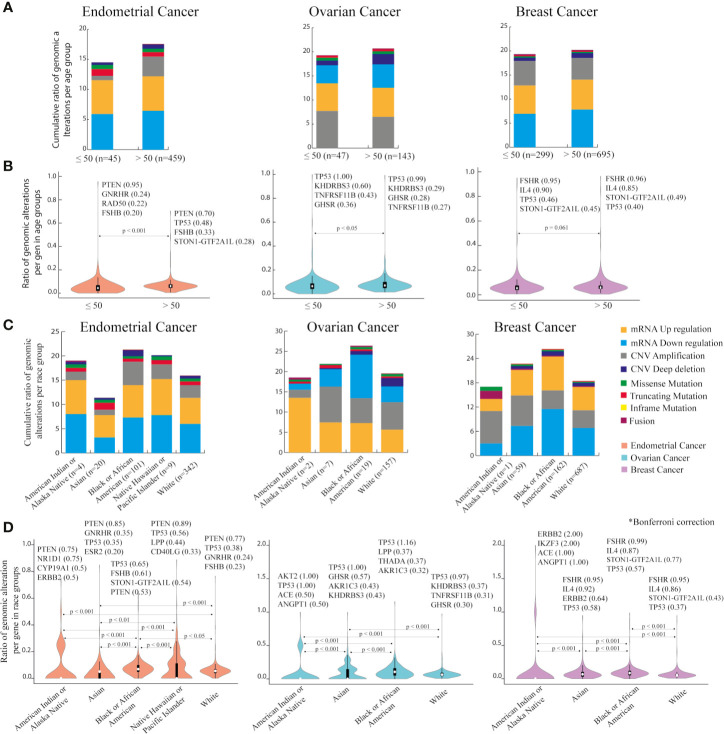
Genomic alterations based on age and race categories in endometrial, ovarian and breast cancer using PanCancer Atlas data in cBioPortal. **(A)** Cumulative ratio of genomic alterations in women aged 50 or less and older than 50 years, per cancer type. **(B)** Ranking of genes with the highest number of genomic mutations per age group in each cancer type. Mann-Whitney U test with significant level of p < 0.05. **(C)** Cumulative ratio of genomic alterations per race category in three cancer types. **(D)** Ranking of genes with the highest number of genomic mutations per race group in the three cancer types. Dunn-Bonferroni *post hoc* method was performed following a significant Kruskal-Wallis test, only significant p-values are presented.

Regarding clinical information of the patients, age and race were evaluated (**Table 2**). As menopause timing is around 50 years old (61), individuals in each cancer type were clustered in two age groups. **Figure 3A** shows the ratio of genomic alterations in the PRG normalized for the number of individuals that match the grouping criteria (**Supplementary Table 10**). mRNA downregulation was the genomic alteration with the highest ratios in EC and BC age groups. CNV amplification was the genomic alteration with the highest ratio in OC. Significantly higher cumulative ratios of genomic alterations were found in women older than 50 years in EC (p < 0.001) and OC (p < 0.05) (**Figure 3B**). *PTEN*, *TP53*, and *FSHR* are highly mutated in EC, OC, and BC age groups respectively.

Considering race categories, individuals classified as Black or African American carried the highest cumulative ratio when compared with others races. This outcome was similar in all cancer types (**Figure 3C**).

**Figure 3D** indicates the genes with the highest amount of genomic alterations per race in EC, OC, and BC. After Bonferroni correction, most of the differences among American Indian or Alaska Native, Asian, Black or African American, White, and Native Hawaiian or other Pacific Islander (only in EC) were significant. In EC, *PTEN* exhibited more mutations in all race categories, except for Black or African American. In OC, *TP53* was by far the most altered gene in all race categories. In BC, *FSHR* was top hit in Asian, Black or African American, and White, whilst *ERBB2* in American Indian or Alaska Native (**Supplementary Table 11**).

#### Pathway Enrichment Analysis

**Figure 4:**
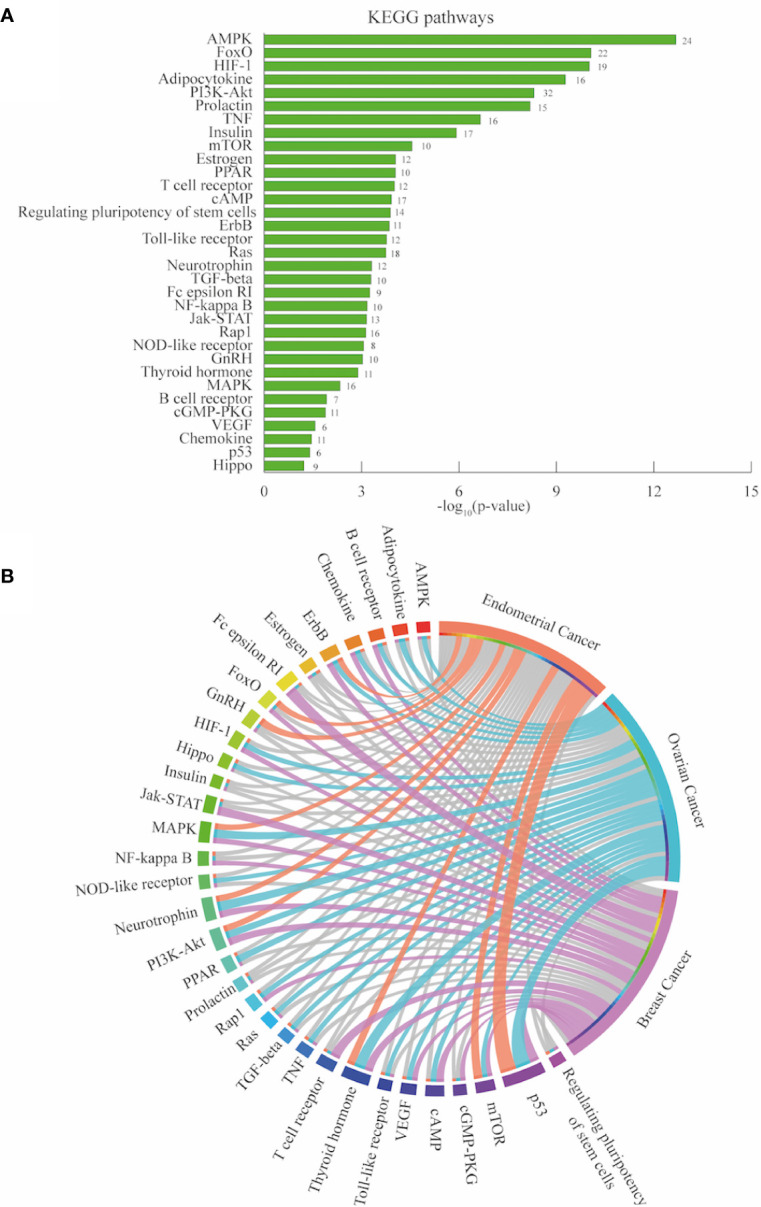
Pathway enrichment analysis endometrial, ovarian and breast cancer. **(A)** Significantly enriched KEGG pathways of associated PCOS related genes (PRG [n=264]) retrieved from DAVID bioinformatics platform. The number in each bar are the gene count per pathways. **(B)** Circos plot depicting the most altered pathways (first quartile colored in each cancer type).

Kyoto Encyclopedia of Genes and Genomes (KEGG) information was accessed from David Bioinformatics Resource to establish the signaling pathways enriched in the 264 PRG gene set (Benjamini-Hochberg - false discovery rate FDR < 0.01) (62, 63). From the 99 terms, diseases terms related to cancer included pathways in cancer, proteoglycans in cancer, EC, among others (**Supplementary Table 13**). Genomic alterations of the genes that integrate 33 signaling pathways were analyzed in each cancer type **Figure 4A**. The circos plot in **Figure 4B** shows that these pathways were genetically more altered in OC than in EC and BC (**Supplementary Table 14**). Jointly, the most altered pathways (first quartile) in the three cancer types were p53, thyroid hormone, neurotrophin, PI3K-Akt, MAPK, mTOR, and ErbB signaling pathways. Being p53 signaling the most altered pathway in EC and OC. On the other hand Fc epsilon RI signaling pathway was the most representative in BC.

#### Transcriptional Levels of PRG in the Gynecological Cancers and BC

Gene expression data in cBioPortal compares mRNA expression z-score relative to all samples. To distinguish tumor specific genes that are up and down regulated from the 264 PRG in EC, OC, and BC, the Gene Expression Profiling Interactive Analysis (GEPIA) web tool was used. GEPIA provides differential gene expression analysis between tumor and normal tissue based on TCGA and GTEx data.

**Figure 5:**
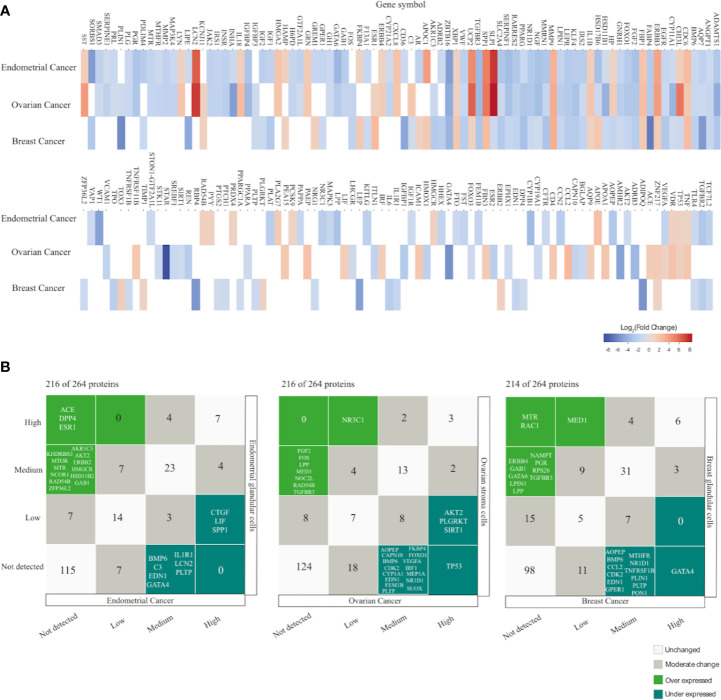
Gene and protein expression profiling of PCOS related genes (PRG) in endometrial, ovarian and breast cancer compared with normal tissue. **(A)** Heatmap displays the differential expressed genes with |Log2FC| = 1, FDR < 0.001 from GEPIA database. Empty spaces indicate absence of differential expression. **(B)** Correlation plot comparing immunohistochemical protein expression profile between cancer samples and healthy tissue according to The Human Protein Atlas (HPA) in each cancer type.

At a transcriptomic level, EC had more altered PRG genes (up and down regulated) with 42.42% (112/264), followed closely by OC with 41.29% (109/264) and lastly BC with 30.68% (81/264). Downregulation was a common feature in the DEG in all cancer types. There were 37 common PRG with mRNA alterations in all cancer types and 134 altered genes in two or at least one cancer type (**Supplementary Table 15**). The top significantly upregulated genes in EC were *SLPI*, *LCN2*, *SPP1*, *UCP2*, *APOC1*; in OC were *SLPI*, *LCN2*, *UCP2*, *CHI3L1*, *SPP1*; in BC were *MMP9*, *ESR1*, *CDC6*, *SPP1*, and *HSD17B6*. In contrast the top down regulated genes in EC were *TGFBR3*, *GATA6*, *WT1*, *ZBTB16*, *SORBS1*; in OC were *ZBTB16*, *AMHR2*, *INHA*, *GATA4*, *STAR*; in BC were *AQP7*, *RBP4*, *ADIPOQ*, *PLIN1*, and *FABP4* (**Figure 5A**).

#### Protein Expression Analysis

The next bioinformatic approach to determine proteins expression changes was attained by exploring The Human Protein Atlas (HPA). The protein profiles generated by microarray-based immunohistochemistry were retrieved from normal and pathological tissue (EC, OC, and BC). HPA resource has analyzed 216 proteins (81.8%) in EC and OC, while 214 (81,1%) proteins in BC. Protein expression was classified as high, medium, low and not detected. Under expression levels were considered either when proteins showed a high/medium expression levels in normal tissue but were not detected in cancer tissue, or when proteins had a high expression levels in normal tissue and low expression levels in cancer tissue. In contrast, over expression levels were considered if proteins had either low or not detected expression levels in normal tissue and high expression levels in cancer tissue or if proteins were not detected in normal tissue but displayed medium expression levels in cancer tissue.

In EC, 25 (11,6%) proteins have altered expression; 15 proteins were under expressed: ACE, DPP4, ESR1, AKR1C3, AKT2, ERBB2, GAB1, HMGCR, HSD11B2, KHDRBS3, MTOR, MTR, NCOR1, RAD54B, ZFP36L2, and 10 were over expressed: CTGF, LIF, SPP1, BMP6, C3, EDN1, GATA4, IL1R1, LCN2, PLTP (**Figure 5B**) (**Supplementary Table 16**).

In OC, 27 (12.5%) proteins displayed altered expression; eight proteins were under expressed in malignant tissue NR3C1, FGF2, FOS, LPP, MED1, NOC2L, RAD54B, TGFBR3 whereas 19 proteins were over expressed TP53, AKT2, PLGRKT, SIRT1, AOPEP, CAPN10, BMP6, CDK2, CYP1A1, EDN1, FEM1B, FKBP4, FOXO1, IRF1, VEGFA, MEP1A, NR1D1, PLTP, SUOX (**Figure 5B**) (**Supplementary Table 17**).

In BC, 25 (11.6%) proteins have altered expression; 12 proteins were under expressed MTR, RAC1, MED1, ERBB4, GAB1, GATA6, LPIN1, LPP, NAMPT, PGR, RPS26, TGFBR3, and 13 were over expressed GATA4, AOPEP, BMP6, CCL2, CDK2, EDN1, GPER1, MTHFR, NR1D1, PLIN1, PLTP, PON1, TNFRSF1B (**Figure 5B**) (**Supplementary Table 18**).

This third omics analysis revealed proteins with altered expression levels (under/over expressed) when comparing normal vs tumor tissue.

#### Identification of PRG Altered at Genomic, Transcriptomic, and Proteomic Level

**Figure 6:**
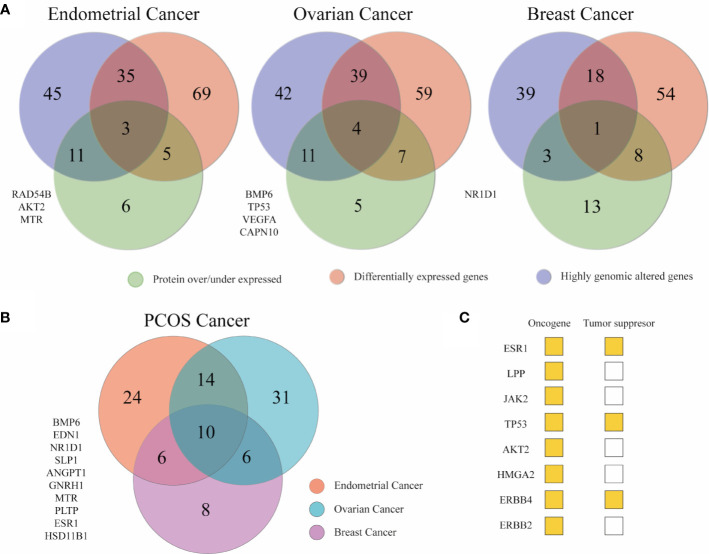
Key genes between gynecological cancer and PCOS. **(A)** Venn diagrams depicting the number of unique and shared associated PCOS across the three omics approaches. The list of genes at the left are the genes that appeared in the three omics approaches in each cancer type. **(B)** The Venn diagram shows PCOS related genes altered in at least two omics approaches in breast and gynecological cancers to stablish a relationship between the syndrome and cancer genetics.

Venn diagrams were used to compile all the results. It was detected 172 genes at least in one approach in EC, 167 in OC, and 136 in BC. Overlapped genes from the three *in silico* analyses are depicted in **Figure 6A** (**Supplementary Table 19**). In **Figure 6B**, the genes that were altered in two approaches in EC, OC, and BC, independently, were merged to obtained a list of 99 genes that correlated PCOS with cancer. Fourteen genes (*ACE*, *LYN*, *LPP*, *JAK2*, *RAD54B*, *TP53*, *INHA, GATA4*, *FKBP4*, *AKT2*, *HMGA2*, *VWF*, *MMP9*, *SST*) were shared between EC and OC. Six genes (*ERBB4*, *CDC6*, *HAMP*, *CHI3L1*, *LPIN1*, *ERBB2*) were common between EC and BC. Other six genes (*AKR1C3*, *FABP4*, *TGFBR3, ZNF217*, *UCP2*, *PLIN1*) were common between OC and BC. Lastly, 10 genes (*BMP6*, *EDN1*, *NR1D1*, *SLPI*, *ANGPT1*, *GNRH1, MTR, PLTP, ESR1, HSD11B1*) were common to all (**Supplementary Table 20**). From the 36 (14 + 6+6+10) aforementioned genes, eight are classified as tumor suppressor or oncogenes according to the Network of Cancer Genes (NCG6.0) (64). To examine the function of these 36 genes, a functional enrichment analysis was conducted using g:profiler (**Supplementary Table 21**). Results showed that these genes were enriched in cell proliferation, response to endogenous stimulus/lipid/hormones, protein phosphorylation, apoptosis, and cell death. Nearly 56% of the 36 genes were active in regulating cell population proliferation. Apart from *TP53*, *GATA4*, *ERBB4*, and *ERBB2* similar genes were involved in response to hormone and response to endogenous stimulus.

#### Contextualization of Bioinformatic Data

Gynecological cancers and BC have been reported in women with PCOS. However the molecular mechanisms underlying this association is not clear. Hence, this study aims to explore the status of strongly PCOS related genes in EC, OC, and BC through oncogenomic databases. Cancer is a genetic disease that arises from genetic alterations that can lead to abnormal downstream effects as dysregulated transcriptional programs (65). The knowledge of protein alteration in malignant transformation is currently growing for the discovery of biomarkers (66). Hereafter, we analyze the genomic alterations, transcriptional profiles, and protein expression changes of PRG in EC, OC, and BC as similarly performed in other studies (67, 68).

The initial exploration of the PRG performed in g:Profiler showed that the most relevant GO:biological processes were response to endogenous stimulus, organic subtract, and hormones as reported previously from 287 DEG in six PCOS data sets (45). In a group of upregulated genes detected in adipose tissue from women with PCOS, the term cell proliferation was enriched similarly as seen in this review report (39). WikiPathway supported the hypothesis that the selected genes are implicated in EC, OC, BC pathways, as well as in pancreatic, bladder, colorectal, and gastric cancer pathways. Subsequently, the most significant Human Phenotype Ontology term was insulin resistance, a significant risk factor for hormone driven women’s cancers (10, 34).

Cancer hallmarks are defined as distinguishing features that explain the cellular properties needed to transform normal to malignant cells in detriment of host tissue (69). Interestingly, 8% of PRG have hallmarks of cancer and cancer driver genes specifically for EC, OC and BC were also found.

The first omics approach aimed to detect genomic status in the PRG compared with PNRG. In all cancer types, the frequency mean of genomic alterations in the PRG was significantly higher than in the PNRG. Thus, the PRG might be associated with cancer risk and allowed further exploration of these genes in oncodatabases.

CNV amplifications in OC and mRNA downregulation in both EC and BC were the most common genomic alterations. In each cancer type, the different ranking of genes according to the number of genomic alterations may have given a hint of relevant genes towards specific cancer prediction in women with PCOS. In EC, PTEN, and TP53 were the genes with the highest number of mutations. PTEN is a tumor suppressor, whose mutations are found in endometroid cancer in combination with microsatellite instability. The fact that PTEN mutations are presented in 20% of endometrial hyperplasia, suggests that mutations in this gene are an initial event in the development of carcinogenesis (70). Mutations in PTEN, PIK3CA, KRAS, and β-catenin are frequently seen in type I endometrial cancers, in contrast TP53, ERBB2, p16, and E-cadherin are seen in type II endometrial cancer (71). PTEN, alongside with other genes involved in insulin signaling pathways, were upregulated in women with PCOS and EC when tested in endometrial tissue (RNA) and serum (protein) (72).

After protein-protein interaction network, TP53 was detected as a hub protein in ovarian endometriosis and PCOS (43). TP53 is highly mutated in high grade serous ovarian carcinoma and seems to be a precursor of ovarian carcinoma. Nonetheless in a mouse model, TP3 mutations were not involved in ovarian tumorigenesis unless PTEN loss was concomitantly found (73).

In breast cancer patients, FSHR was expressed in endothelial cells and blood vessels at the tumor edge. Intriguingly, blood vessels expressing FSHR were associated with invasive tumors. What is more FSHR might participate in tumor vascular network remodeling, as tumors are surrounded an organized microvessels distribution contrary to an heterogenous vascular network in normal tissue (32).

Regarding age groups, women with EC and OC over 50 years of age have significantly higher number of mutations than younger women. This is expected as mutations accumulate with age due to cellular senescence. However, it was reported that pre-menopausal women with PCOS (< 54 years old) are at a higher risk of EC and OC (14). This corroborates that PCOS is not exclusively caused by genetic events, it has several risk factors such as obesity, IR, hormonal impairments, that should be considered when evaluating cancer risk.

The wide range of PCOS prevalence (3–20%) has been attributed to geographical location, race, ethnicity, or diagnose criteria applied (74). Even symptom frequency such as hirsutism, acne, insulin resistance, and obesity varies with ethnicity (4). This phenotypic variance can be due to genetic background composition. Similarly gynecological cancer and BC risk in women with PCOS fluctuated according to the geographical locations of the patients sampled and diagnosis criteria considered (14). Hence, after racial analysis in EC, OC, and BC, it was found significant differences in the ratio of genomic alterations in race groups, being Black and African American the race with higher number of genomic alterations in EC, OC, and BC, with a significant Bonferroni correction of p < 0.05. In age and race group, the genomic alterations distribution and the top genomic mutated genes were in accordance with the results already mentioned before grouping.

KEGG pathway enrichment analysis was conducted considering the genomic alterations of the 264 PRG. Pathways with the highest amount of genomic alterations were p53 in EC and OC, and Fc epsilon RI in BC. P53 signaling pathway participates in cell cycle arrest, programmed cell death, DNA repair, inhibition of angiogenesis, and cellular senescence (75). Fc epsilon RI signaling pathway promotes immune cells degranulation, cytokine/chemokine release, and inflammation mainly in the presence of allergens. Abundance of immune cells expressing Fc epsilon RI have been associated with favorable breast cancer prognosis. Histological images analysis evidenced that degranulation have a tumor cytotoxic effect (76). Cooperatively, p53, thyroid hormone, neurotrophin, PI3K-Akt, MAPK, mTOR, ErbB signaling pathways were in the first quartile of altered pathways in all cancer types. Thyroid hormone signaling pathway mediates physiological process involving growth, embryonic development, differentiation, and metabolism (77). Neutrophin signaling pathway plays a role in the survival, development and function of neural cells (78). PI3K-Akt is a signal transduction pathway involved in cell cycle regulation, apoptosis, transcription, protein synthesis, and cancer progression of certain gynecological tumor (79). mTOR signaling pathways participates in lipid metabolism, protein synthesis, and cytoskeletal organization. PI3K-AKT coupled with the downstream activation of mTOR pathways form a signaling network often altered in cancer as ovarian malignancies. Inhibitors of this network are being tested as a therapeutic strategy in ovarian cancer (80). MAPK signaling pathway also controls cell cycle, differentiation, and inflammation and is associated with oncogenesis and drug resistance (81). Finally, ErbB signaling pathway initiates with the activation of receptor tyrosine kinases and continues with the activation of other pathways: Akt and MAPK. ErbB signaling pathway regulates cell proliferation, migration, angiogenesis, and metastasis in several cancers (82).

The second omics approach focused on PRG expression profiling matching tumor versus normal tissue based on RNA-sequencing data in the GEPIA website. Comparison between normal and cancer cells contributes to the identification of tumor genes and its oncogenic functions (83). Our results indicate that 30% to 40% of PRG were differentially expressed and most of them were down regulated. This and other studies have noticed that most of the DEG were commonly down regulated when PCOS was compared with either OC and EC (43, 84). Huiyu et al. (85) proposed 53 key genes in obese insulin resistant women with PCOS and breast cancer, selecting PCOS DEG that have a prognostic effect in breast cancer. None of the 53 genes were found in our DEG, presumably as PRG were not selected according to cancer prognostic effect, but to its relationship with the syndrome. A list of 94 genes was proposed by Atiomo et al. (84) after comparing RNA sequencing data in obese women with PCOS (n=1) and women with EC (n=1) relative to healthy endometrium, of them 5 were differentially expressed in EC in our study (IGF2, ERBB4, SLPI, CYP1B1, F13A1). The fact that 37 out of 264 PRG have a transcriptional alteration in all cancer types, suggests there is a common mechanism or pathways acting in them.

The third omics approach addressed proteins expression changes. It has been shown that proteome profiling of relevant tissue helps to develop biomarkers for diagnosis and premature detection (86). Our results showed that most proteins have the same expression pattern in normal and cancer tissue, of them several were undetected. However, there were 11% to 12% of proteins in EC, OV and BC that displayed discordant expression between normal and tumor tissue based on immunohistochemistry according to HPA.

After merging the omics data, EC had more altered genes in at least one omics approach (172) followed by OC (167) and BC (136). This is in accordance with higher endometrial cancer risk in women with PCOS. The genes altered in at least two omics approaches in EC, OC, and BC were integrated to produce a list of 99 genes. From them 26 genes were highly altered at a genomic, transcriptomic and proteomic level in at least one pair of cancer types and 10 genes were common for all. A recent publication reported a total of 141 critical genes involved in EC, OC, and BC and 52 common genes when cervical cancer was added. This indicates there is a common mechanism of oncogenesis in women’s cancers, most likely related to hormone regulation (87). From the list of 141 genes previously published, 18 (ESR1, TP53, MMP9, ERBB2, LCN2, WT1, IGF2, SPP1, CYP1B1, IGF1R, APOE, FOXO1, CCL2, TNF, VEGFA, PGR, PTGS2, AR) appeared in our 99 gene list. Functional analysis of the 36 overlapped genes between PCOS and cancer (26 + 10) revealed its key action in cell proliferation, response to endogenous and hormones as reported in women’s cancers (87).

The evidence provided in our study suggests that women with PCOS are at risk of cancer development, yet further experimental studies are required to validate our findings. This study contributes towards the determination of molecular markers in women with PCOS, that may confer them a risk for cancer development. The identification of molecular and clinical data enhancing oncogenesis will be favorable for better cancer risk estimates, preventative treatment as well as early diagnosis. For instance women with EC and BC treated at stage 1, have an improvement of 5-survival rate (12). The accumulation of omics information will accelerate the understanding of molecular basis of this complex endocrinopathy and its association with cancer.

## Pharmacogenomics in PCOS

There has been a growing interest in single nucleotide polymorphism (SNPs) and their potential for predicting individual drug response. In the near future with pharmacogenomics advances, it may be possible to prescribe specific drugs with precise dosage based on the SNP profile reducing toxicity and boosting treatment efficacy (88).

The pharmacological treatment of women with PCOS is mainly oriented to lessen metabolic abnormalities and to restore fertility. The commonly prescribed drugs include metformin, clomiphene citrate (CC), oral contraceptives pills (OCP), follicle stimulating hormone (FSH) among others. Drug resistance, side effects, undefined dosage for favorable results and toxicity in offspring has been reported in treated women with PCOS (89). Having a collection of SNPs conferring PCOS susceptibility and drug response will contribute for developing a gene score risk prediction and personalized treatments.

### Drug Treatment in PCOS

Metformin is an insulin-sensitizer and its mechanism of action is through the activation of AMP-activated protein kinase (AMPK). AMPK activation in the liver is catalyzed by the serine-threonine kinase (STK11), in other words STK11 acts as a mediator and not metformin target. *In vitro STK11* knock out experiments in mice resulted in AMPK deactivation, production of adipocytes and enhance lipogenic gene expression (90). The organic cation transporter 1 (*SLC22A1*) facilitates metformin uptake in hepatocytes meanwhile the multidrug and toxin extrusion protein 1–2 (*SLC47A1* and *SLC47A2*) and the organic cation transporter 2 (*SLC22A2*) mediate its hepatic excretion and elimination into urine. All transporters are expressed in ovaries, hepatocytes and muscles, which are relevant tissues in PCOS (91). *In vitro* and *in vivo* assays have shown an inverse relationship between glucose levels and OCT proteins expression in diabetic context (92, 93).

Metformin reduces glucose absorption in the intestine and hepatic glucogenesis by inhibiting respiratory complex chain, moreover it increases glucose uptake in muscle and liver cells for glucose serum clearance (94). It also stimulates the hepatic synthesis of SHBG and regulates lipid metabolism by suppressing lipogenesis and by promoting β-oxidation (95). In women with PCOS metformin has demonstrated to improve menses frequency, ovulation, conception, and weight reduction (89). Its side effect is related to gastrointestinal discomfort (96).

CC is an antagonist competitor of 17β-estradiol. When CC binds to estrogen receptors in the hypothalamus, it stimulates gonadotropin-releasing hormone (GnRH) from the pituitary gland, which in turn, leads to the production of follicle stimulating hormone (FSH) and luteinizing hormone (LH). These hormones aid in ovarian follicle maturation and ovulation (97). CC has been widely used in the treatment of infertility in women with PCOS, nevertheless it has a negative effect on cervical mucus and endometrium. It has been associated with thromboembolic events and multiple gestations (98, 99).

OCP are used in the treatment of menstrual irregularities and hyperandrogenism in women with PCOS. There are two types of OCP: those containing estrogen and progestogen (combined pills), and the other ones with progestogen only. Estrogen content increases sex hormone binding globulin (SHBG), hence decreasing free circulation androgens. Progestogen suppresses LH secretion and restrains androgen production by ovarian and adrenal glands. It also competes with androgen receptor and prevents subsequent androgen actions (89, 100). After 6 months of OCP treatment, testosterone, glucose, and LH levels were reduced, and hirsutism improvements were seen. However a slight increase of lipids was also detected (101, 102). OPC have potential adverse effects on IR, diabetes mellitus and coagulability (89), indeed Maier et al. (101) stated OCP is suitable to moderate hirsutism and amenorrhea in women with PCOS, who do not have metabolic comorbidities.

Gonadotropins (i.e. exogenous FSH) are administrated in the case of reported CC resistance (103). They promote follicle growth, selection of dominant follicle, ovulation and augment the possibilities of fertilization. Their action is mediated through its specific receptor (FSHR) located in granulosa cells in the ovary. Their main drawback is drug dosage, since it can cause various follicle development, hyperstimulation syndrome and multiple pregnancies at high doses (104).

Medical treatment in PCOS patients is dependent on several traits such as age, BMI or hormone profile. For instance aging has been positively associated with better response to CC, explained by an increase of baseline FSH level in older women (105). High BMI is a negative predictor for ovulation induction with CC, presumably because fat tissue can alter its pharmodynamics (97). In contrast, high BMI is a consistent predictor of positive metformin response, as OCT proteins are expressed in adipose tissue (106). Women with PCOS, who have elevated Anti-Müllerian hormone (AMH) levels are CC resistant or they may require higher CC starting dosage (107).

There is no standard protocol to treat PCOS patients, nevertheless monotherapy or combination drug therapy studies have been published. Legro et al. (108) has reported a significantly better outcome when combining metformin and CC, evidenced in a higher ovulation rate. On the other hand, Pedersen et al. (96) showed weight reduction but an increase of triglycerides when combination therapy with metformin and OCPs was used.

Despite efforts to use monotherapy or combination therapy to improve PCOS symptoms, responses are variable and the determinants of this inconsistency remain elusive. Variability in PCOS efficacy treatment has been speculated to be associated to gene-treatment interactions (96). Apart from drug response variability, 30% women with PCOS did not respond to metformin (94) and 15% to 40% are CC resistance after receiving 150 mg/day (99). There is evidence that genes polymorphisms might be predictors for drug resistance, emphasizing the relevance of pharmacogenomics studies in PCOS.

### Key Pharmacogenomics Findings

Key characteristics of the 13 pharmacogenetic studies published in PCOS are summarized in **Tables 3**–**5**. There were polymorphisms associated with baseline traits in the Caucasian population. López-Bermejo et al. (90) found *STK11* as a marker of poor metabolic profile. Schweighofer et al. (94) recognized that OCTs might play a role in glucose metabolism, independently of metformin treatment, as insulin levels (measured by C-peptide) were higher in individuals with OCTs SNPs. It has been hypothesized that the components of glucose metabolism are substrates of OCTs (**Table 3**).

**Table 3:** Single nucleotide polymorphism associated with traits before drug ingestion.

Drug	Duration	Reference SNPs	Genes	Effect	Cohort origin or ethnicity/PCOS criteria	No. PCOS patients	Ref.
Metformin 850 mg/d at dinner time	12 months	rs8111699	*STK11*	G allele was associated with higher insulin and IGF-I levels (p < 0.005).	Caucasian Northern Spanish girls/NS	85 (36 PCOS)	(90)
NA	NA	rs12208357 rs34447885 rs34104736 rs683369 rs34059508 rs36103319 rs628031	*SLC22A1*	Higher C-peptide levels at baseline and after glucose load found in patients with at least one mutant allele in *SLC22A1* and *SLC22A2* polymorphisms (p < 0.05). This hold true in a subsequent grouping among PCOS lean group (p =0.007).	Austria Caucasians/Rotterdam criteria	422	(94)
		rs316019	*SLC22A2*				
		rs11212617	*ATM*				

NA, not available; NS, not stated.

**Table 4:** Studies with clinical improvements due to drug treatment.

Drug	Duration	Reference SNPs			Genes	Effect	Cohort origin or ethnicity/PCOS criteria	No. PCOS patients	Ref.
Metformin 500 mg 3 times a day plus diet	6 months	rs1801278			*IRS1*	Lower fasting glucose 17α-OHP and AS was detected (p<0.05).	NS/Rotterdam criteria	60	(109)
Metformin 1000 - 2700 mg/d plus a low calorie diet	6 months	rs12208357 rs34130495 rs34059508 rs72552763			*SLC22A1*	Body weight drop menstrual cyclicity improvements, increased SHBG levels and FAI, glucose and insulin levels reduction (p<0.05).	Italy Caucasian/Rotterdam criteria	150	(95)
Metformin 500 mg 3 times a day	6 months	rs316019			*SLC22A2*	Insulin levels were reduced (p < 0.001) and G/I ratio was increased (p= 0.001).	Taiwan Asian/NS	87	(110)
CC 50 mg/d and dose raising in 50 mg/d each cycle only up to 150 mg/d	NS	rs6166			*FSHR*	Higher FSH level (p=0.003) and lower BMI range (p=0.039) induced ovulation on any dose.	92% Caucasian, 3% Asian, 4% Black/Rotterdam criteria	193	(97)
CC 100 mg/d	1 cycle	EM *1/*1 *1/*2 *2/*2 *1/*10	EM *1/*49 *1/*52 *2/*10 *1/*5	IM *10/*10 *10/*41 *10/*49 *5/*10	*CYP2D6*	Absence of ovulation after the first cycle treatment correlated with lower (E)-clomiphene (active metabolite to induce ovulation) concentration (p=0.036).	Korean Asian/NS	42 (19 PCOS)	(107)
a) Metformin 1000 mg twice a day b) Metformin with OCP (150 µg DSG + 30 µg EE)	12 months	rs12208357 rs72552763			*SLC22A1*	Combined medication was associated with weight reduction (p < 0.001) and increased triglycerides (p < 0.01).	Caucasian/Rotterdam criteria	40	(96)
		rs2289669 rs2252281			*SLC47A1*				
		rs12943590			*SLC47A2*				
		rs11212617			*ATM*				
		rs1169288 rs2464196			*HNF1A*				
a) OCP (20 µg EE + 75 µg GSD) b) OCP plus 100 mg/d spironolactone in hirsute women	6 months	rs2414096			*CYP19*	There was an increase in lipids profile and SHBG. Reduction of testosterone levels FAI, DHEAS, AS, hirsutism score and a mild decline in systolic blood pressure, LH levels and fasting glucose was reported (p < 0.05).	95% were Caucasian 5% mixed descent/Rotterdam criteria	162 51 (treated of them 32 showed hirsutism)	(102)
a) OCP (20 μg EE + 75 μg of GSD) b) OCP plus 100 mg/d spironolactone hirsute women	6 months	rs3763676			*HSD17B5*	Reduction in systolic blood pressure glucose, DHEAS, AS, hirsutism score, testosterone levels, FAI and LH levels and an increase in lipids and SHBG was indicated (p<0.05).	95% Caucasian, 5% African European descent/Rotterdam criteria	49	(101)
a) Metformin 500mg with increments until 1000mg twice a day b) CC 50mg/d (50 mg increment each cycle until 150 mg in poor responders) c) Metformin and CC	30 weeks or before pregnancy	rs741765			*STK11*	Ovulation rate per cycle or per patient in the metformin group was lower than in the other 2 treatments (p < 0.001). The mean number of ovulations per subject was higher with combined treatment (p < 0.001).	NS/Rotterdam criteria	312	(108)
		rs2234693			*ESR1*				
		rs1934963 rs1799853 rs3892097			*CYP2C9*				
		D19S884			*FBN3*				

17α-OHP, 17a-hydroxyprogesterone; AS, androstenedione; G/I, glucose/insulin; SHBG, sex hormone binding globulin; FAI, free androgen index; EM, extensive metabolizers; IM, intermediate metabolizers; DSG, desogestrel; EE, ethinyl estradiol; GSD, gestodene; DHEAS, dehydroepiandrosterone sulfate; LH, luteinizing hormone; CC, clomiphene citrate; OCP, oral contraceptive pills; NS, not stated.

**Table 5:** Associated SNPs with clinical difference in drugs response.

Drug	Duration	SNPs	Genes	Effect	Cohort origin or ethnicity/PCOS criteria	No	Ref.
Metformin 500 mg 3 times a day plus diet	6 months	rs1801278	*IRS1*	G allele was associated with lower fasting insulin levels LH levels and insulin resistance (p<0.001),. DHEAS and total testosterone concentrations were reduced in G allele carries (p<0.05) while increased with A allele.	NS/Rotterdam criteria	60	(109)
Metformin 850 mg/d at dinner time	12 months	rs8111699	*STK11*	G/G genotype had strong metabolic improvements (lower insulin, IGF-1, FAI, lipids, fat mass and abdominal mass), G/C had intermediate response, C/C had almost no response (p< 0.005).	Northern Spain, Caucasian/NS	85 (36 PCOS)	(90)
Metformin 1000 - 2700 mg/d plus a low calorie diet	6 months	rs12208357 rs34130495 rs34059508 rs72552763	*SLC22A1*	Carriers of wild a type allele in all positions had total cholesterol and triglycerides reduction after treatment (p=0.006).	Caucasian Italian/Rotterdam criteria	150	(95)
Metformin 500 mg 3 times a day	6 months	rs683369 rs628031	*SLC22A1*	rs683369 G allele carriers (p < 0.001) and rs628031 A allele carries (p = 0.001) showed an increased insulin sensitivity (higher G/I ratio)	Taiwan Asian/NS	87	(110)
CC 50 mg/d and dose raising in 50 mg/d each cycle only up to 150 mg/d	Not available	rs6166	*FSHR*	G/G genotype carriers were resistant to clomiphene citrate compared other genotypes (P < 0.05).	92% Caucasian 3% Asian 4% Black and 1% unknown/Rotterdam criteria	193	(97)
10– 450 IU of rFSH	IVF duration	rs6165	*FSHR*	Heterozygous genotype patients showed higher response (lower ratio of FSH dose/number of retrieved oocytes) to exogenous FSH (p < 0.05).	Caucasian Italian/NS	40	(104)
a) Metformin 500mg and increasing until 1000mg twice a day b) CC 50mg/d (50 mg increment each cycle until 150 mg in poor responders) c) Metformin and CC	30 weeks or before pregnancy	rs8111699	*STK11*	In the Met-only group the rs8111699 C allele (CC or CG) was associated with a decreased ovulation per cycle/per patient compared with G/G genotype (p<0.01).	NS/Rotterdam criteria	312	(108)
a) 50 mg/d of CC in absence of ovarian response 100–150 mg/d next cycles. b), In case of CRA, 75 IU of rFSH was used and ½ dose was increased daily if follicle <10 mm	4.2 - 6.7 months	rs6166	*FSHR*	CRA was noticed in PCOS patients with G/G genotype (p=0.05). Same result was obtained in a the pool analysis (p= 0.03) when compared with other genotypes.	Netherlands Caucasian Discovery cohort/WHO-II group of anovulatory subfertility women Replicated cohort/ Rotterdam criteria	Discovery cohort 159 Replicated Cohort 185 PCOS	(103)
**Cohort 1** Metformin 500 mg/d, with dose increasing by 500 mg every 2 weeks to a final dose of 1500 mg/d **Cohort 2** 2000 mg/d of metformin **Cohort 3** 2000 mg/d of metformin	**Cohort 1** 3 months **Cohort 2** 9 months **Cohort 3** 30 weeks or before pregnancy	rs683369	*SLC22A1*	rs683369 G allele was associated with less weight loss in cohort 1 but was not replicated in other cohorts.	**Cohort 1** Caucasian (57%) African–American (21%),, Asian (11%) and mixed ethnicity (11%) **Cohort 2** Caucasian (46%), African–American (50%), Asian (4%) **Cohort 3** Caucasian (71%), African–American (16%) Asian (2%), Native American (11%) Rotterdam criteria (cohort 1-2) NIH criteria (cohort 3)	**Cohort 1:** 38 PCOS **Cohort 2:** 26 PCOS **Cohort 3:** 131 PCOS	(91)
		rs11212617	*ATM*				
		rs2252281 rs2289669 rs8065082	*SLC47A1*				

LH, luteinizing hormone; DHEAS, dehydroepiandrosterone sulfate; IGF-1, insulin-like growth factor 1; FAI, free androgen index; G/I, glucose to insulin; FSH, follicle-stimulating hormone; CC, clomiphene citrate; IVF, in vitro fertilization; NS, not stated; rFSH, recombinant FSH; CRA, clomiphene-resistant anovulation.

Other studies detailed in **Table 4**, failed to detect gene-drug interactions, but they confirmed therapies efficiency in terms of body composition, endocrine, metabolic and ovulation improvements (95, 96, 101, 102, 108–110). Besides, ovulation predictors such as high FSH levels and low BMI range were identified (97). A pharmacokinetics exploration of CC proved that women who did not ovulate show lower concentrations of the active metabolites (107).

Based on the findings mainly in Caucasian population *STK11*, *SLC22A1*, *FSHR* are potential candidate genes with SNPs that may predict drug response or resistance to commonly prescribed drugs for PCOS treatment (90, 91, 95, 97, 103, 104, 108, 110). For instance, the best responders to exogenous FSH, (rs6165; heterozygous G/A) will need lower dosage for ovarian stimulation whereas higher dosages will induce severe ovarian hyperstimulation syndrome (104). Homozygous PCOS carriers of G/G *FSH* polymorphism (rs6166) are less likely to restore ovulatory menstrual cycles under CC treatment, consequently they should be treated with exogenous gonadotropins (i.e., rFSH) (103). Alternative allele homozygosity of rs8111699 SNP (G/G) showed robust metabolic improvements after metformin treatment and reduced therapy duration (90) (**Table 5**).

**Figure 7:**
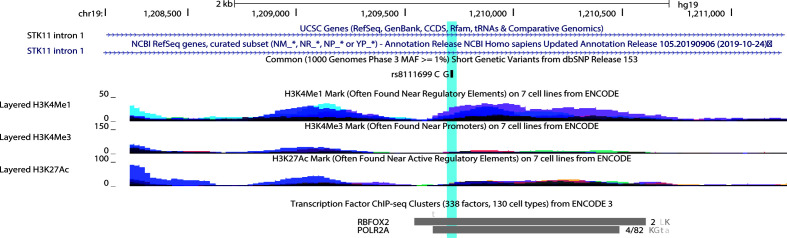
UCSC Genome Brower (Human Feb 2009 (GRCh37/hg19 assembly) displaying rs8111699 (highlighted) and tracks representing histone marks and transcription factors in the first intron of *STK11*. For histone marks peak height is proportional to the signal amplitude with colors representing databases in seven different cell lines*. For transcription factor binding tracks, the length of the box indicates region of occupancy and the darkness is proportional to the signal strength observed in several cell lines. To the right there is the number of cell types contributing to the cluster or a fraction that corresponds the number of cell where the factor was detected out of all cell assayed. The letters represent the cell abbreviation L, HepG2; K, K562; a, adrenal gland; G, GM12878; t, tibial nerve. * 7 Cell lines for histone marks from ECODE: GM12878 (B-lymphocyte, lymphoblastoid), H1-hESC (embryonic stem cells), HSMM (skeletal muscle myoblasts), HUVEC (umbilical vein endothelial cells), K562 (erythroleukemic), NHEK (epidermal keratinocytes), NHLF (lung fibroblasts).

Most of the polymorphisms associated with drug response are missense mutations, except for an intronic nucleotide change in *STK11*. According to UCSC genome browser rs8111699 occurred in the region of H3K4me1signal and H3K2Ac, frequently found near active regulatory elements. Besides, the binding site of POLR2A and RBFOX2 transcription factors occurs within the SNP region (111). RBFOX2 occupied the marked region in HepG2 and K562 cell lines, while adrenal gland, GM12878, K562, and tibial nerve contribute to the POL2A cluster (**Figure 7**). POLR2A is part of the largest subunit of RNA polymerase II and has modifications to recruit other factors that regulate transcription, mRNA processing and chromatin state. RBFOX2 is a RNA-binding protein that regulates alternative splicing and seems to act as a coregulatory factor of estrogen receptor alpha (ER-α) (112). According to HaploReg V4 database, rs8111699 is classified as a regulatory element in liver tissues by the 25-state model with an open chromatin region confirmed by DNase assay (**Table 6**). SNPs in LD (r^2^ ≥ 0.8) with rs8111699 were investigated to find a potential functional variant on a regulatory region. Although all proxy SNPs were located in non-coding regions, they had a promoter or enhancer activity in the target tissues.

**Table 6:** Bioinformatic characterization of SNPs in linkage disequilibrium with rs8111699 in European Population (HaploReg V4).

Locus	ReferenceSNPs	LD (r2) EUR	Chromatin states^a^		H3K4me1^b^	H3K4me3^c^	H3K27a^d^	H3K9ac^e^	Dnase
			Adult liver (AL)	HepG2 (H)					
5 kb 5' of STK11	rs7253626	0.9	–	–	–	–	–	–	
1.6kb 5' of STK11	rs7254997	0.97	EnhW2	EnhAF	AL, H	AL	AL, H	AL, H	–
intronic	rs7256801	1	TxReg	TxReg	AL	AL, H	AL, H	AL, H	H
intronic	rs12611000	0.95	TxEnh5	TxEnh5	AL, H	AL, H	AL, H	AL, H	–
intronic	rs8111699	1	TxEnh5	TxReg	AL, H	H	AL, H	AL, H	H
intronic	rs7259033	0.92	TxReg	TxEnh5	AL, H	AL, H	AL	AL, H	H
intronic	rs8106285	0.87	–	–	AL	–	AL	AL	–
intronic	rs34928889	0.81	–	–	–	–	AL	–	–
intronic	rs11084889	0.94	–	–	–	–	AL	–	–
intronic	rs60977562	0.96	–	–	AL	–	AL	–	–
intronic	rs60490879	0.96	–	–	–	–	–	–	–
intronic	rs7253853	0.95	–	–	–	–	–	–	–
3'-UTR	rs10415095	0.92	–	–	–	–	–	–	–

EUR, European population; LD, linkage disequilibrium.

a Chromatin states based on 25-state model: EnhW2, Weak Enhancer 2; EnhAF, Active Enhancer Flank; TxReg, Transcribed & regulatory (promoter/enhancer); TxEnh5, Transcribed 5' preferential and Enhancer.

b H3K4me1 is a histone mark associated with enhancers and DNA regions downstream of transcription starts.

c H3K4me3 is a histone mark associated with promoters that are active or poised to be active.

d H3K27ac is a histone mark that indicates active enhancers, promoters or active transcription sites.

e H3K9ac is a histone mark connected with active promoters.

### Absence of Agreement in PCOS Pharmacogenomics

The lack of consensus on the role of genetics and drug metabolism in PCOS can be explained as follows. Certain polymorphisms might have an effect on metabolic parameters even prior to therapy, as demonstrated by López-Bermejo et al. (90) and Schweighofer et al. (94). Parameters to assess treatment effectiveness are various such as increase ovulation (97, 103, 108), number of oocytes retrieved (104) or clinical responses (weight loss, lipids profiles or oral glucose tolerance tests) (110). Some studies combined PCOS patients with other women sharing similar traits as hyperinsulinemia, androgen excess, ovulatory dysfunction, to increase statistical power (90, 103, 107). Confounding factors such as diet or lifestyles were not cautiously supervised in all studies, except for two studies that added a low calorie diet as part of the treatment (95, 109). Drug dosages, duration and timing are inconstant, so it is hard to draw definite conclusions about gene-drug interaction. Another factor to consider is drug-drug interaction in combination therapy, as noted by Legro et al. (108). When metformin and CC were administrated, a significant association with C/C genotype in rs2234693 (*ESR1*) and higher ovulation per cycle were discovered, but the same outcome did not exist in the CC treated group.

Conflicting results in metformin transport proteins polymorphism (i.e. *SLC22A1*, *SLC22A2, SLC47A1*, and *SLC47A2*) and drug response could be explained as metformin glucose-lowering effects is restricted to the gastrointestinal tract (113). Other aspect worth mentioning is that allele frequencies may differ between ethnic groups, thus making the association less apparent, depending on the population studied. Population allele frequencies of the identified SNPs associated with drug response, according to the 1000 Genomes Project (phase 3) are shown in **Table 7** (114). Pau et al. (91) did not show differences in metformin response parameters such as fasting glucose levels, testosterone levels, and ovulatory rate between ethnic subgroups in three American cohorts. These results need to be addressed cautiously as dosage and duration of treatment are not equal in the compared cohorts. Even though pharmacogenetics will help to predict *a prior* drug efficacy, the applicability of this knowledge may not be directly transferred between diverse ethnics groups due to different genetic composition (115).

**Table 7:** Allele frequencies for relevant genetic variants associated with PCOS treatment response in human populations worldwide.

Gene	Reference SNP	Discovery cohort	Consequence	WT > M	Human Populations							
					Caucasians		Latin American		Asian		African	
*IRS-1*	rs1801278 (Gly971Arg)	NS	Missense mutation	C > G,T*	Finland	0.06	Colombia	0.03	China	0.01	Barbados	0.07
					Great Britain	0.04	Mexico	0.02	Japan	0.05	USA	0.06
					Spain	0.15	Peru	0.02	Vietnam	0.01	Gambia	0.04
					Italia	0.08	Puerto Rico	0.05	Bangladesh	0.05	Nigeria	0.11
*STK11*	rs8111699	Caucasian	Intron mutation	C* > G	Finland	0.50	Colombia	0.45	China	0.01	Barbados	0.32
					Great Britain	0.51	Mexico	0.58	Japan	0.01	USA	0.42
					Spain	0.55	Peru	0.39	Vietnam	0.01	Gambia	0.27
					Italia	0.51	Puerto Rico	0.49	Bangladesh	0.33	Nigeria	0.44
*SLC22A1*	rs12208357 (Arg61Cys)	Caucasian	Missense mutation	C > T*	Finland	0.06	Colombia	0.04	China	0.00	Barbados	0.02
					Great Britain	0.06	Mexico	0.02	Japan	0.00	USA	0.02
					Spain	0.05	Peru	0.01	Vietnam	0.00	Gambia	0.00
					Italia	0.06	Puerto Rico	0.02	Bangladesh	0.02	Nigeria	0.00
*SLC22A1*	rs34130495 (Gly401Ser)	Caucasian	Missense mutation	G > A*	Finland	0.02	Colombia	0.01	China	0.00	Barbados	0.01
					Great Britain	0.02	Mexico	0.01	Japan	0.00	USA	0.02
					Spain	0.03	Peru	0.01	Vietnam	0.00	Gambia	0.00
					Italia	0.02	Puerto Rico	0.01	Bangladesh	0.01	Nigeria	0.00
*SLC22A1*	rs34059508 (Gly465Arg)	Caucasian	Missense mutation	G > A*,C	Finland	0.01	Colombia	0.02	China	0.00	Barbados	0.00
					Great Britain	0.04	Mexico	0.04	Japan	0.00	USA	0.00
					Spain	0.02	Peru	0.02	Vietnam	0.00	Gambia	0.00
					Italia	0.01	Puerto Rico	0.01	Bangladesh	0.00	Nigeria	0.00
*SLC22A1*	rs72552763	Caucasian	Inframe deletion	ATGAT > AT*	Finland	0.16	Colombia	0.27	China	0.01	Barbados	0.06
					Great Britain	0.21	Mexico	0.37	Japan	0.00	USA	0.07
					Spain	0.16	Peru	0.38	Vietnam	0.02	Gambia	0.03
					Italia	0.20	Puerto Rico	0.18	Bangladesh	0.12	Nigeria	0.03
*SLC22A1*	rs683369 (Leu160Phe)	Asian	Missense mutation	C > A, G*,T	Finland	0.19	Colombia	0.19	China	0.12	Barbados	0.04
					Great Britain	0.23	Mexico	0.05	Japan	0.13	USA	0.05
					Spain	0.24	Peru	0.04	Vietnam	0.14	Gambia	0.00
					Italia	0.17	Puerto Rico	0.13	Bangladesh	0.15	Nigeria	0.00
*SLC22A1*	rs628031 (Met408Val)	Asian	Missense mutation	G > A*,C	Finland	0.49	Colombia	0.35	China	0.22	Barbados	0.23
					Great Britain	0.39	Mexico	0.12	Japan	0.19	USA	0.29
					Spain	0.43	Peru	0.10	Vietnam	0.25	Gambia	0.32
					Italia	0.34	Puerto Rico	0.26	Bangladesh	0.39	Nigeria	0.23
*FSHR*	rs6166 (Ser680Asn)	Caucasian	Missense mutation	T > C*	Finland	0.50	Colombia	0.44	China	0.30	Barbados	0.37
					Great Britain	0.44	Mexico	0.34	Japan	0.34	USA	0.43
					Spain	0.43	Peru	0.41	Vietnam	0.32	Gambia	0.35
					Italia	0.46	Puerto Rico	0.48	Bangladesh	0.36	Nigeria	0.48
*FSHR*	rs6165 (Ala307Thr)	Caucasian	Missense mutation	C > G,T*	Finland	0.50	Colombia	0.56	China	0.67	Barbados	0.26
					Great Britain	0.56	Mexico	0.67	Japan	0.64	USA	0.38
					Spain	0.57	Peru	0.66	Vietnam	0.65	Gambia	0.20
					Italia	0.53	Puerto Rico	0.48	Bangladesh	0.63	Nigeria	0.23

^a^Frequency of the minor allele marker with *, NS, not stated; WT, wild type; M, mutant allele.

### Future Perspectives in PCOS Pharmacogenetics

Although there are several reported prescribed drugs for women with PCOS, the number of pharmacogenomics studies are limited. Larger and ethnically diverse trials are required to confirm the existing results and to possibly tailor a treatment based on the women’s genetic screening.

This endocrinopathy is a multifactorial polygenic disorder, thus not only pathological features but their response to treatment could be partly attributed to complex genetic basis. Future research can be focused on building a gene score combining metformin transporters polymorphisms in order to detect a stronger collective effect in drug response, as performed by Díaz et al. (116) when investigating metformin in androgen excess patients. Polymorphisms in *SLC2A2*, that encodes glucose transporter (117), or in *SP1* and *PPAR-α*, which are transcription factors of metformin transporters (118) could be further analyzed in metformin response. Future research that explores dosage, treatment duration, ethnicity, circadian drug effect, drug pharmacokinetics and pharmacodynamics in women with PCOS will upgrade their treatment.

## Final Remarks

This study explored 264 strongly related PCOS genes (PRG) to identify key genetic factors involved in endometrial, ovarian and breast oncogenesis using *in silico* approaches. The fact that genomic alterations in PRG were significantly higher compared with a set of non-diseases genes in all cancer types, allowed us to explore PRG in other databases. Transcription dysregulation was detected in the gynecological cancers and breast cancer relative to normal tissue, being downregulation a common feature among the DEG. Less than 30 proteins displayed altered expression in all cancer contexts. Using an overlapping analysis, 26 genes were highly altered between two cancer types. Ten genes were identified to be involved in the three cancer types analyzed. The identification of 36 genes involved in cell proliferation regulation and response to hormone, support their association with oncogenesis in these hormone driven cancers.

The identification of key gene polymorphisms that may influence the response to commonly prescribed drug in women diagnosed with PCOS, will guide the selection of medication to enhance efficacy and reduce side effects. Up to now, 10 SNPs in four genes are reported to be predictors of drug response. These outcomes are promising, but more research is needed to design a treatment algorithm based on patient’s clinical history and genomic profile.

## Author Contributions

VY conceived the study, analyzed the data, and wrote the manuscript. AL-C, SG, and JG-C gave valuable conceptual and scientific advices. AP-V made substantial contribution with manuscript writing. AL-C, AP-V, IY, SG, JG-C, IA-C, PG-R, AKZ, PEL, and AKZ contributed to the structure and design of the manuscript. CP-y-M supervised the project. All authors contributed to the article and approved the submitted version.

## Funding

The research reported in this publication was supported by Universidad UTE.

## Conflict of Interest

The authors declare that the research was conducted in the absence of any commercial or financial relationships that could be construed as a potential conflict of interest.
