# Supplementary material for: Oncology and Pharmacogenomics Insights in Polycystic Ovary Syndrome: An Integrative Analysis
Source: Front Endocrinol (Lausanne). 2020 Oct 26;11:585130. doi: 10.3389/fendo.2020.585130 (PMC7729301; doi:10.3389/fendo.2020.585130)
Supplement: Supplementary file 1 [file DataSheet_1.pdf]

Supplementary Data

Bioinformatic Exploration Methods

Gene Sets

A total of 923 PCOS related genes (PRG) were collected from the databases DisGeNET https://www.disgenet.org/ (1), Ensembl http://www.ensembl.org (2) PCOSKB http://pcoskb.bicnirrh.res.in/gene.php (3) DISEASES https://diseases.jensenlab.org/Search (4) and literature review (5-13). Open Targets Platform (https://www.targetvalidation.org/) was used to obtain a list of the strongly related PCOS genes based on the overall association score that ranges from 0 to 1 (the stronger evidence of an association the higher the value) (14). Further analysis was carried out with a list of 264 genes (score ≥0.05).

PCOS non-related genes (PNRG) were retrieved from a list of 4.957 non-diseases genes (15). After excluding genes associated with PCOS, reproductive diseases, endometrial/ovarian/breast cancer in Open Targets Platform, 1728 genes were left. Randomly 300 genes were selected from the previous list for the following analysis.

The driver gene set per cancer type were taken from the recent study conducted by (16).

Enrichment map of associated PCOS genes

For functional enrichment analysis with the 264 PRG, g:Profiler database (https://biit.cs.ut.ee/gprofiler/gost) was accessed to acquire significant features (FDR < 0.001) linked to gene ontology (Biological Process), biological pathways (Reactome, WikiPathways) and human phenotype ontology (17).

Cancer Hallmarks

The Catalogue of Somatic Mutations in Cancer (COSMIC) contains gene functional annotations that trigger cancer, condensed in 10 cancer hallmarks. To discover if the 264 PRG can be catalogued as having a role in cancer, COSMIC resource was examined (18)

Genomic Alteration in TCGA PanCancerAtlas (PCA)

Genomic alterations (amplification, deep deletion, mRNA upregulation, mRNA downregulation, missense mutation, truncating mutation, inframe mutation and fusion gene) in PRG, PNRG and driver genes were interrogated in Uterine Corpus Endometrial

Carcinoma (n=507 complete samples), Ovarian Serous Cystadenocarcinoma (n=201), Breast Invasive Carcinoma (n=994) through the cBioPortal (http://www.cbioportal.org/) (19,20). mRNA expression profiles z-score (± 2) were calculated relative to all samples. Mutations codified with unknown significance were not considered. For PRG and PNRG gene set statistics normalization considered numbers of genes examined.

The clinical annotations selected were diagnosis age and race. For clinical data comparison within each cancer type normalization contemplated the number of patients in each category (ratio). The ratio and percentage of genetic alterations per age and race group were calculated, with this data the ranking of genes and categories with the greatest number of all genetic alterations were determined. Regarding age: 45, 47, 299 women aged less or 50 years and 459, 143, 695 age more than 50 years old in endometrial, ovarian and breast cancer respectively. In the race group: 4, 2, 1 individuals were American Indian or Alaska Native; 20, 7, 59 were Asian; 101, 19, 162 were Black or African American; 342, 157, 687 were White in endometrial, ovarian and breast cancer respectively. Only in endometrial cancer the 9 individuals were Native Hawaiian or Other Pacific Islander.

Kruskal Wallis-test with Bonferroni correction was performed in python to detect significant differences of frequency in all genetic alterations among gene set and race categories, while Mann–Whitney U test for age groups statistics.

KEGG Pathways enrichment analysis of associated PCOS genes: David Bioinformatics Resources website (https://david.ncifcrf.gov/summary.jsp) was used to obtain unified data from KEGG (21,22). The enrichment analysis of signaling pathways was carried out in the 264 PRG genes, considering terms with a significant FDR < 0.01. Then, to identify the most perturbated signaling pathways in each cancer type. The number genetic alterations of the genes in each signaling pathway were added and normalization took into account the number of genes in the pathways and the individuals in each cancer type.

Gene expression analysis

The website Gene Expression Profiling Interactive Analysis (GEPIA, http://gepia2.cancer-pku.cn/#degenes) provides tumor vs. normal differential gene expression analysis among other functions based on TCGA and GTEx RNA-seq data

(23). The search contents and thresholds in Breast invasive carcinoma (BRCA), Ovarian serous cystadenocarcinoma (OV) and Uterine Corpus Endometrial Carcinoma (UCEC) dataset were set as follows: |Log2FC| Cutoff: 1.0, q-value Cutoff: 0.01, LIMMA for differential method.

Protein expression analysis

Protein profiling in normal and human tumor tissue based on immunohistochemisty using tissue microarrays is available in Human Protein Atlas (HPA, https://www.proteinatlas.org/) portal (24,25). Therefore comparisons among protein expression levels (high, medium, low and non-detected) of the 264 PRG between normal and cancer tissues were performed. Protein expression level of normal tissue were taken from endometrium glandular cells, ovarian stroma cell and breast glandular cells.

Characterization of overlapped genes

To investigate if the genes commonly altered in at least 2 of the cancer types are cataloged as oncogenes and/or tumor suppressor genes, the Network of Cancer Genes (NCG6.0) database was examined. It has a list of 711 known cancer genes with their respective annotations (26). General functions of the 31 genes obtained in this study were investigated using g:profiler with the settings previously mentioned.

References

1. Piñero J, Ramírez-Anguita JM, Saüch-Pitarch J, Ronzano F, Centeno E, Sanz F, et al. The DisGeNET knowledge platform for disease genomics: 2019 update. Nucleic Acids Res. 2020 Jan 8;48(D1):D845–55.

2. Yates AD, Achuthan P, Akanni W, Allen J, Allen J, Alvarez-Jarreta J, et al. Ensembl 2020. Nucleic Acids Res. 2020 Jan 8;48(D1):D682–8.

3. Joseph S, Barai RS, Bhujbalrao R, Idicula-Thomas S. PCOSKB: A KnowledgeBase on genes, diseases, ontology terms and biochemical pathways associated with PolyCystic Ovary Syndrome. Nucleic Acids Res. 2016 Jan 4;44(D1):D1032–5.

4. Pletscher-Frankild S, Pallejà A, Tsafou K, Binder JX, Jensen LJ. DISEASES: Text mining and data integration of disease–gene associations. Methods. 2015 Mar 1;74:83–9.

5. Chen Z-J, Zhao H, He L, Shi Y, Qin Y, Shi Y, et al. Genome-wide association study identifies susceptibility loci for polycystic ovary syndrome on chromosome 2p16.3, 2p21 and 9q33.3. Nat Genet. 2011 Jan;43(1):55–9.

6. Shi Y, Zhao H, Shi Y, Cao Y, Yang D, Li Z, et al. Genome-wide association study identifies eight new risk loci for polycystic ovary syndrome. Nat Genet. 2012 Sep;44(9):1020–5.

7. Pau C, Saxena R, Welt CK. Evaluating reported candidate gene associations with polycystic ovary syndrome. Fertil Steril. 2013 May 1;99(6):1774–8.

8. Lee H, Oh J-Y, Sung Y-A, Chung H, Kim H-L, Kim GS, et al. Genome-wide association study identified new susceptibility loci for polycystic ovary syndrome. Hum Reprod. 2015 Mar 1;30(3):723–31.

9. Day F, Hinds DA, Tung JY, Stolk L, Styrkarsdottir U, Saxena R, et al. Causal mechanisms and balancing selection inferred from genetic associations with polycystic ovary syndrome. Nat Commun. 2015 Sep 29;6(1):1–7.

10. Shim U, Kim H-N, Lee H, Oh J-Y, Sung Y-A, Kim H-L. Pathway Analysis Based on a Genome-Wide Association Study of Polycystic Ovary Syndrome. PLOS ONE. 2015 Aug 26;10(8):e0136609.

11. Zhang X-Z, Pang Y-L, Wang X, Li Y-H. Computational characterization and identification of human polycystic ovary syndrome genes. Sci Rep. 2018 Aug 28;8(1):1–7.

12. Day F, Karaderi T, Jones MR, Meun C, He C, Drong A, et al. Large-scale genome-wide meta-analysis of polycystic ovary syndrome suggests shared genetic architecture for different diagnosis criteria. PLOS Genet. 2018 Dec 19;14(12):e1007813.

13. Zhang Y, Ho K, Keaton JM, Hartzel DN, Day F, Justice AE, et al. A genome-wide association study of polycystic ovary syndrome identified from electronic health records. medRxiv. 2019 Dec 15;2019.12.12.19014761.

14. Carvalho-Silva D, Pierleoni A, Pignatelli M, Ong C, Fumis L, Karamanis N, et al. Open Targets Platform: new developments and updates two years on. Nucleic Acids Res. 2019 Jan 8;47(D1):D1056–65.

15. Chakraborty S, Panda A, Ghosh TC. Exploring the evolutionary rate differences between human disease and non-disease genes. Genomics. 2016 Jul 1;108(1):18–24.

16. Dietlein F, Weghorn D, Taylor-Weiner A, Richters A, Reardon B, Liu D, et al. Identification of cancer driver genes based on nucleotide context. Nat Genet. 2020 Feb;52(2):208–18.

17. Raudvere U, Kolberg L, Kuzmin I, Arak T, Adler P, Peterson H, et al. g:Profiler: a web server for functional enrichment analysis and conversions of gene lists (2019 update). Nucleic Acids Res. 2019 Jul 2;47(W1):W191–8.

18. Tate JG, Bamford S, Jubb HC, Sondka Z, Beare DM, Bindal N, et al. COSMIC: the Catalogue Of Somatic Mutations In Cancer. Nucleic Acids Res. 2019 Jan 8;47(D1):D941–7.

19. Cerami E, Gao J, Dogrusoz U, Gross BE, Sumer SO, Aksoy BA, et al. The cBio Cancer Genomics Portal: An Open Platform for Exploring Multidimensional Cancer Genomics Data. Cancer Discov. 2012 May 1;2(5):401–4.

20. Gao J, Aksoy BA, Dogrusoz U, Dresdner G, Gross B, Sumer SO, et al. Integrative Analysis of Complex Cancer Genomics and Clinical Profiles Using the cBioPortal. Sci Signal. 2013 Apr 2;6(269):pl1–pl1.

21. Huang DW, Sherman BT, Lempicki RA. Systematic and integrative analysis of large gene lists using DAVID bioinformatics resources. Nat Protoc. 2009 Jan;4(1):44–57.

22. Huang DW, Sherman BT, Lempicki RA. Bioinformatics enrichment tools: paths toward the comprehensive functional analysis of large gene lists. Nucleic Acids Res. 2009 Jan 1;37(1):1–13.

23. Tang Z, Li C, Kang B, Gao G, Li C, Zhang Z. GEPIA: a web server for cancer and normal gene expression profiling and interactive analyses. Nucleic Acids Res. 2017 Jul 3;45(W1):W98–102.

24. Uhlén M, Fagerberg L, Hallström BM, Lindskog C, Oksvold P, Mardinoglu A, et al. Tissue-based map of the human proteome. Science. 2015 Jan 23;347(6220).

25. Uhlen M, Zhang C, Lee S, Sjöstedt E, Fagerberg L, Bidkhori G, et al. A pathology atlas of the human cancer transcriptome. Science. 2017 Aug 18;357(6352).

26. Repana D, Nulsen J, Dressler L, Bortolomeazzi M, Venkata SK, Tourna A, et al. The Network of Cancer Genes (NCG): a comprehensive catalogue of known and candidate cancer genes from cancer sequencing screens. Genome Biol. 2019 Jan 3;20(1):1.
